# Enterotype *Bacteroides* Is Associated with a High Risk in Patients with Diabetes: A Pilot Study

**DOI:** 10.1155/2020/6047145

**Published:** 2020-01-22

**Authors:** Jiajia Wang, Wenjuan Li, Chuan Wang, Lingshu Wang, Tianyi He, Huiqing Hu, Jia Song, Chen Cui, Jingting Qiao, Li Qing, Lili Li, Nan Zang, Kewei Wang, Chuanlong Wu, Lin Qi, Aixia Ma, Huizhen Zheng, Xinguo Hou, Fuqiang Liu, Li Chen

**Affiliations:** ^1^Department of Endocrinology, Qilu Hospital of Shandong University, Jinan, Shandong, China 250012; ^2^Institute of Endocrine and Metabolic Diseases of Shandong University, Jinan, Shandong, China 250012; ^3^Key Laboratory of Endocrine and Metabolic Diseases, Shandong Province Medicine & Health, Jinan, Shandong, China 250012

## Abstract

**Background:**

More and more studies focus on the relationship between the gastrointestinal microbiome and type 2 diabetes, but few of them have actually explored the relationship between enterotypes and type 2 diabetes. *Materials and Methods.* We enrolled 134 patients with type 2 diabetes and 37 nondiabetic controls. The anthropometric and clinical indices of each subject were measured. Fecal samples of each subject were also collected and were processed for 16S rDNA sequencing. Multiple logistic regression analysis was used to determine the associations of enterotypes with type 2 diabetes. Multiple linear regression analysis was used to explore the relationship between lipopolysaccharide levels and insulin sensitivity after adjusting for age, BMI, TG, HDL-C, DAO, and TNF-*α*. The correlation analysis between factors and microbiota was identified using Spearman correlation analysis. The correlation analysis between factors was identified using partial correlation analysis.

**Results:**

Gut microbiota in type 2 diabetes group exhibited lower bacterial diversity compared with nondiabetic controls. The fecal communities from all subjects clustered into two enterotypes distinguished by the levels of *Bacteroides* and *Prevotella.* Logistic regression analysis showed that the *Bacteroides* and *Bacteroides* and *Prevotella* enterotype. Partial correlation analysis showed that lipopolysaccharide was closely associated with diamine oxidase, tumor necrosis factor-alpha, and Gutt insulin sensitivity index after adjusting for multiple covariates. Furthermore, the level of lipopolysaccharide was found to be an independent risk factor for insulin sensitivity.

**Conclusions:**

We identified two enterotypes, *Bacteroides* and *Prevotella*, among all subjects. Our results showed that the *Bacteroides* enterotype was an independent risk factor for type 2 diabetes, which was due to increased levels of lipopolysaccharide causing decreased insulin sensitivity.*Bacteroides* and *Prevotella* enterotype. Partial correlation analysis showed that lipopolysaccharide was closely associated with diamine oxidase, tumor necrosis factor-alpha, and Gutt insulin sensitivity index after adjusting for multiple covariates. Furthermore, the level of lipopolysaccharide was found to be an independent risk factor for insulin sensitivity. *Bacteroides* and

## 1. Introduction

Type 2 diabetes (T2D), which is a complex disorder influenced by both genetic and environmental components, has become a major public health issue throughout the world. Nowadays, increasing evidence shows the relationship between gut microbiota and T2D [1–5]. Many studies revealed that the microbiome of type 2 diabetic patients is characterized by an enrichment in several opportunistic pathogens [2], sulphate-reducing bacteria [6, 7], and the depletion of some probiotics [8] and some butyrate-producing bacteria [2, 6, 7]. In addition, both human and rodent studies have shown that the transfer of the intestinal microbiota can also result in the transfer of specific metabolic disease phenotypes [9, 10], involving hyperglycemia. Modification of the gut microbiota by diet and prebiotics can improve glucose tolerance and insulin response [11, 12]. Moreover, the therapeutic effects of some hypoglycemic agents such as metformin and *α*-glucosidase inhibitors may partially be mediated by the gut microbiota [13, 14]. However, the outcomes are not always concordant. Larsen et al. found a lower within-sample diversity in T2D patients [1] while Qin et al. did not observe a significant difference in this diversity between T2D patients and the control group [2]. Also, the latter study found an increase in *Akkermansia muciniphila*, a mucus-colonizing bacterium that plays a protective role in the gut barrier function, in T2D patients. However, Zhang et al. reported the opposite effect, specifically, a decreased abundance of *A. muciniphila*, in diabetic and glucose-intolerant patients [15]. These discrepancies show that we still have much to know about the relationship between T2D and gut microbiota.

Enterotype is another means to investigate the gut microbiota. Arumugam et al. first introduced this method in 2011 in a study where based on the taxonomic composition, they clustered human fecal metagenomic samples from three continents into three enterotypes: the *Bacteroides* enterotype (ET B), *Prevotella* enterotype (ET P), and *Ruminococcus* enterotype which are not associated with ethnicity, gender, age, or body mass index (BMI) [16]. Since then, several studies have replicated enterotypes in new datasets and to different extents, both in the numbers of enterotypes and strength of the statistical support [17–21]. However, few studies have directly investigated the relationship between enterotypes and T2D. Hence, here, using taxa and enterotype data, we aimed to investigate if enterotypes exist within T2D and nondiabetic controls and if there is an association between enterotypes and T2D.

## 2. Materials and Methods

### 2.1. Participants

Originally, 150 type 2 diabetic patients and 50 nondiabetic controls were included. All 150 T2D patients were diagnosed using the World Health Organization diagnostic criteria for diabetes [22]. The plasma glucose concentration of all 50 nondiabetic controls was evaluated by a fasting oral glucose tolerance test (OGTT). The exclusion criteria for our study were as follows: (1) history of inflammatory bowel diseases, (2) persistent diarrhea, or (3) use of antibiotics, or use of probiotic or prebiotic supplements within three months before the data collection. Among T2D patients, nine used antibiotics within three months before fecal collection, one had persistent diarrhea, and six were using prebiotic supplements. These patients were excluded and the final T2D patient number was 134 (65 females, 69 males). Among the nondiabetic controls, six drank yogurt within three days before fecal collection and seven did not finish OGTT. Thus, the final number in the control group was 37 (27 females, 10 males). Informed consent was obtained from all of the participants. This study was approved by the ethical committee of the Qilu Hospital of Shandong University (IRB no. KYLL-2017-595).

### 2.2. Diet and Medicine

All T2D patients were investigated during their hospitalization in the Qilu Hospital of Shandong University. Nondiabetic controls were people who worked in the Qilu Hospital of Shandong University. All participants answered questionnaires about their diet history. They all ate Chinese food in their daily life and there was no vegetarian among them.

The hypoglycemic medication usage history of T2D patients within half a year before the fecal collection was asked and recorded.

### 2.3. Anthropometric Measurements

The height, weight, and blood pressure (BP) of each subject were measured using standard protocols. Body mass index (BMI) was calculated as weight (kg) divided by height squared (m^2^). BP was measured three consecutive times using a BP monitor (OMRON, model HEM-752, Fuzzy, Omron Company, Dalian, China) on the left arm of the patient after the patient was sitting for at least five minutes. The average reading was used for analysis.

### 2.4. Serum Biochemistry

After at least 10 hours of overnight fasting, venous blood samples were collected for the measurement of fasting plasma glucose (FPG), high-density lipoprotein cholesterol (HDL-C), and triglyceride levels (TGs) using an automatic analyzer (Architect cil6200 Integrated System, Abbott, USA). Fasting insulin (FINS) and fasting C peptide (FCP) were measured using immunochemiluminometric assays (Centaur, Bayer Germany), while glycated hemoglobin (HbA1c) was measured by high-performance liquid chromatography using an HbA1c automatic analyzer (G7, Tosoh, Japan). A 2-hour postprandial plasma glucose (2hPG), 2-hour postprandial plasma insulin (2hINS), and 2-hour postprandial C peptide (2hCP) were measured after subjects completed a 75 g OGTT, using the same method as fasting. Serum diamine oxidase (DAO), lipopolysaccharide (LPS), and tumor necrosis factor-alpha (TNF-*α*) levels were quantified using ELISA (Cusabio Biotech Company, Wuhan, Hubei Province, China; catalog no. CSB-E09945h, CSB-E10137h, and CSB-E04740h, respectively) using blood collected at fasting status. The homeostasis model assessment of *β*-cell function (HOMA-*β*), and insulin resistance (HOMA-IR) was calculated using FPG and FCP [23]. The Gutt-insulin sensitivity index (Gutt-ISI) was calculated as follows: Gutt‐ISI = [75000 + (G0 − Gl20) × 18 × 0.19 × BW]/{120 × lg[(I0 + I120)/2] × [(G0 + Gl20) × 90] [G0: FPG (mmol/l), G120: 2hPG (mmol/l), BW: body weight (kg), I0: FINS (uIU/ml), and I120: 2hINS (uIU/ml)].

### 2.5. Fecal Sample Collection

Fecal and blood samples were collected on the same morning. After at least 10 hours of overnight fasting and blood sample collecting, everyone was given a clean plastic plate and a clean tube in which to put and collect stool samples using the toilet in the hospital. Fecal samples were kept at 4°C immediately after defecation and were transported to the laboratory within 12 hours after defecation and stored at −80°C until analysis.

### 2.6. DNA Extraction and PCR Amplification

Microbial DNA was extracted from 171 samples using a QIAamp DNA Stool Mini Kit (Qiagen, Hilden, Germany). The concentration of genomic DNA in each fecal sample was quantified using a NanoDrop 2000 spectrophotometer (Thermo Scientific, Waltham, MA, USA). The DNA integrity and size were assessed by resolving the DNA on a 1% agarose gel via agarose gel electrophoresis.

### 2.7. 16S rRNA Gene Amplicon and Sequencing

Universal primers (341F and 806R) linked with indices and sequencing adaptors were used to amplify the V3-V4 regions of the 16S rRNA gene. The amplicons were sequenced using an Illumina HiSeq platform to obtain 250-bp pair-end reads.

Tags, trimmed of barcodes and primers, were further checked on their rest lengths and average base quality, and 16S tags were restricted to 220–500 bp, such that the average Phred score of the bases was no worse than 20 (Q20).

### 2.8. Statistical Analysis

The copy number of the tags was enumerated and the redundancy of repeated tags was removed. Only the tags with a frequency of more than 1, which tend to be more reliable, were clustered into operational taxonomic units (OTUs), each of which had a representative tag. OTUs were clustered with 97% similarity using UPARSE (http://drive5.com/uparse/), and chimeric sequences were identified and removed using USEARCH (version 7.0). Each representative tag was assigned to a taxa by the RDP classifier (http://rdp.cme.msu.edu/) against the RDP database (http://rdp.cme.msu.edu/) using a confidence threshold of 0.8. The OTU profiling table and alpha/beta diversity analyses were obtained using python scripts of QIIME. The continuous variables with normal distribution were expressed as the mean ± standard deviation (SD), and the variables with nonnormal distribution were presented as the median (interquartile range). The categorical variables were presented as numbers (%). The normal distribution of the data was tested using the Kolmogorov-Smirnov test. Differences between groups were detected using Student's *t* test (for normally distributed continuous variables), the Mann–Whitney *U* test (for skewed continuous variables), or the chi-squared test (for categorical variables). Multiple logistic regression analysis was used to determine the associations of enterotypes with T2D. Multiple linear regression analysis was used to explore the relationship between LPS levels and insulin sensitivity. The correlation analysis between indices and genera was identified using Spearman correlation analysis. The correlation analysis between several indices was identified using partial correlation analysis. Statistical analyses were performed using the Statistical Package for the Social Sciences (SPSS), version 17.0, and package R for enterotype analyses (cluster package). In all statistical tests, *P* < 0.05 was considered significant.

## 3. Results

### 3.1. Clinical Characteristics

The descriptive characteristics of all participants are shown in Table 1. Compared with the nondiabetic controls, T2D patients had significantly higher age, HbA1c, FPG, FINS, 2hPG, 2hINS, and TG. Patients with T2D also exhibited a significantly lower DBP, 2hCP, HDL-C, HOMA-*β*, and Gutt-ISI relative to the nondiabetic control group (all *P* < 0.05). However, there was no difference between the two groups in the HOMA-IR values, which may have been due to the poor *β*-cell function in the T2D group.

### 3.2. Gut Microbiota Analysis between the Control and T2D Groups

#### 3.2.1. Validity Evaluation of Intestinal Microbiota Data

After applying quality control and trimming, we obtained 9,459,608 high-quality clean reads from 171 subjects. The number of clean reads varied between the subjects, from 30,154 to 64,929, with a mean of 55,319.35 (SD 7667.47) ([Supplementary-material supplementary-material-1]). The sequencing depth was sufficient to achieve our study objectives ([Supplementary-material supplementary-material-1]). The sequence length of the trimmed reads was 240–460 bp ([Supplementary-material supplementary-material-1]), and most reads ranged from 400 to 440 bp ([Supplementary-material supplementary-material-1]). The quality of the reads obtained was reliable.

#### 3.2.2. OTU Analysis

The total number of OTUs obtained from 171 subjects was 1141 ([Supplementary-material supplementary-material-1]). Since the number of reads among the subjects had a wide range, we equalized the dataset to avoid deviation, and then the equalized parameter was determined by the sequencing depth. We then randomly extracted 26,437 reads from every sample. The final number of OTUs analyzed was 1129, which accounted for 98.95% of the total OTUs ([Supplementary-material supplementary-material-1]). From this final number of OTUs, 697 were common to both the control and T2D groups, 406 were specific to only the T2D group, and 26 were specific to only the control group ([Supplementary-material supplementary-material-1]).

The RDP classifier was able to assign all of the OTUs at the kingdom level, and 1085 (96.10%) were assigned at the phylum level ([Supplementary-material supplementary-material-1]). OTUs were distributed among 16 bacterial phyla. The four dominant phyla were *Firmicutes* (46.87%), *Bacteroidetes* (35.05%), *Proteobacteria* (11.05%), and *Actinobacteria* (4.54%) ([Supplementary-material supplementary-material-1]). Furthermore, 1036, 1018, 904, and 612 of the OTUs were further assigned at the class, order, family, and genus level, respectively ([Supplementary-material supplementary-material-1]).

#### 3.2.3. Alpha and Beta Diversity Analysis

**Figure 1:**
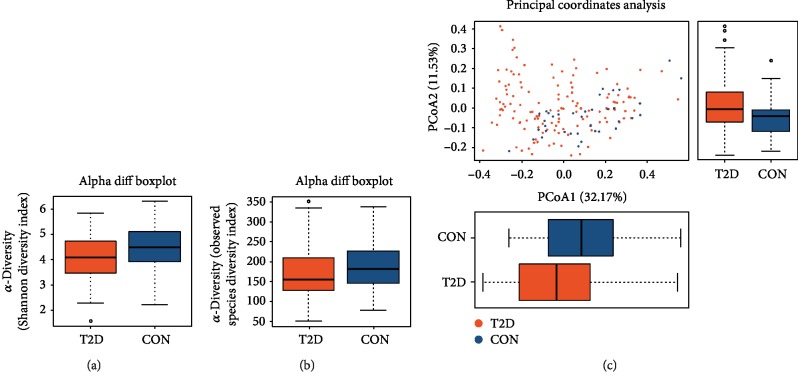
The *α*-diversity and *β*-diversity analysis of the T2D and control groups. (a) The Shannon index difference between the T2D and control groups. *P* = 0.009. (b) The observed species index difference between the T2D and control groups. *P* = 0.040. The *x*-axes of both (a) and (b) show the two different groups. The *y*-axes show the *α*-diversity index value. Abnormal values are shown by “o.” (c) PCoA of the microbiota between the T2D and control groups. The *x*-axis represents the first principal coordinate, and the percentage represents the effect on the difference of the two groups. The *y*-axis represents the second principal coordinate, and the percentage represents the effect on the difference of the two groups. *P*_*x*_ = 0.001, *P*_*y*_ = 0.005. The bottom solid horizontal line represents the minimum value, the lower dotted vertical line represents the first quartile, the center solid horizontal line represents the median, the upper dotted vertical line represents the third quartile, and the top solid horizontal line represents the maximum value. The orange color represents the T2D group. The blue color represents the control group. CON: control group; PCoA: principal coordinates analysis.

Alpha (*α*) diversity was used to evaluate species variety in the samples. Analysis of the *α*-diversity, based on the Shannon index and observed species index, revealed that there was a lower bacterial diversity in the fecal samples from T2D patients compared with the nondiabetic control group (Figures 1(a) and 1(b), [Supplementary-material supplementary-material-1]). The beta (*β*) diversity—the composition of the microbiota—between the control and T2D groups was analyzed using principal coordinates analysis (PCoA), and the results showed a significant difference between the control and T2D groups (Figure 1(c)).

#### 3.2.4. Differential Analysis

**Figure 2:**
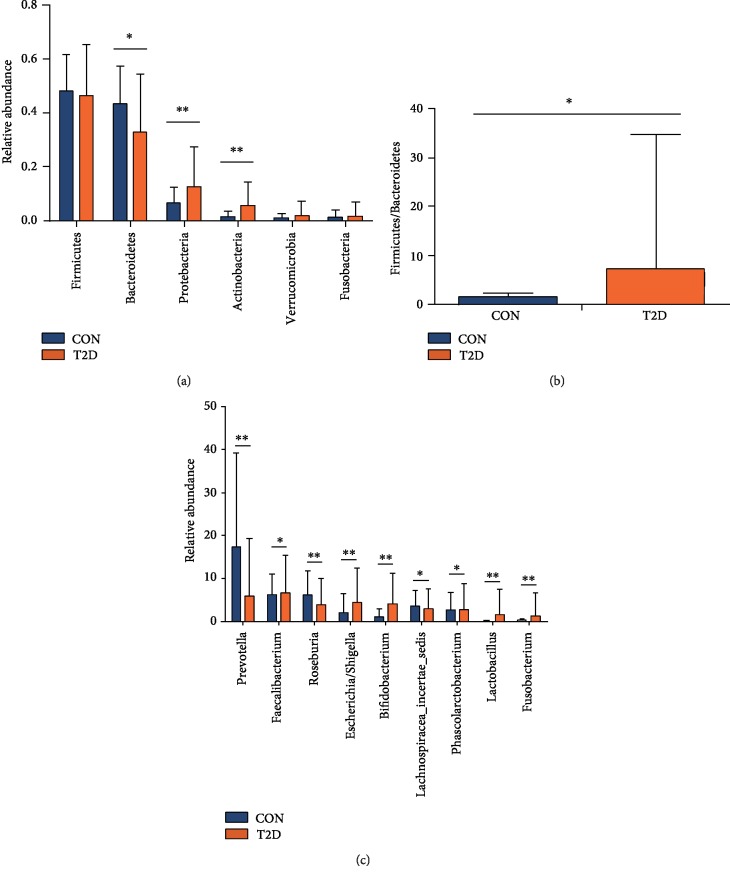
Relative abundances of fecal taxa at different levels. (a) For differences in the fecal microbiota at the phylum level. (b) The F/B ratio in two groups. (c) For differences in the fecal microbiota at the genera level. Statistical analysis was performed by Mann–Whitney *U* test. ^∗^*P* < 0.05, ^∗∗^*P* < 0.01. CON: nondiabetic control group; T2D: type 2 diabetes group.

We examined the structure of the gut microbiome in two groups. At the phylum level, we found that the T2D group had lower numbers of Bacteroidetes and a higher F/B ratio than the control group. In addition, the T2D group was enriched for Proteobacteria and Actinobacteria (Figures 2(a) and 2(b)). At the genus level, there were 23 genera significantly different between the two groups ([Supplementary-material supplementary-material-1]). Among them, there were 9 genera for which the abundance was within the top 20 (Figure 2(c)).

#### 3.2.5. Factor Analysis

The levels of DAO, LPS, and TNF-*α* were compared between the T2D and control group. Excluding all hemolytic samples, a total of 144 samples were analyzed (108 T2D and 36 nondiabetic). After analysis, we found that the T2D group had significantly higher levels of DAO, LPS, and TNF-*α* than the control group ([Supplementary-material supplementary-material-1]).

### 3.3. Enterotype Analysis of All 171 Subjects

#### 3.3.1. Cluster Numbers and Characteristics of Enterotypes

**Figure 3:**
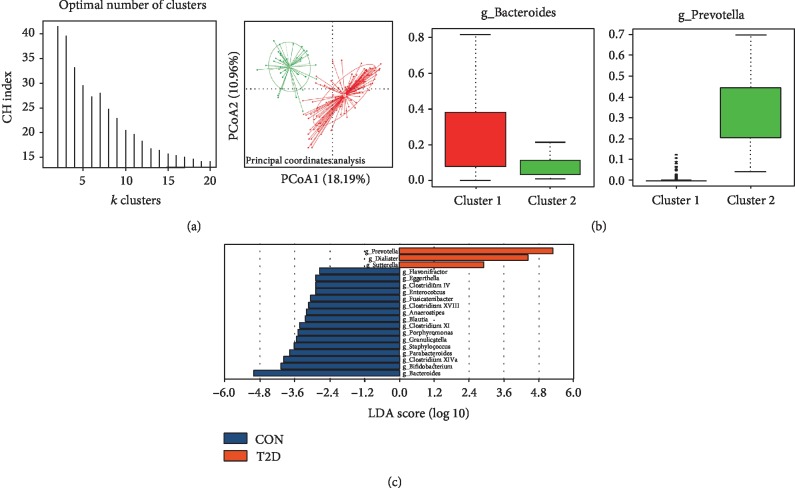
Enterotypes identified in 171 participants using PCoA. (a) Panel (A) shows that the data are most naturally separated into two clusters via LefSe (LDA EffectSize) analysis. The *x*-axis shows the cluster number, and the *y*-axis shows the Calinski-Harabasz index, which is a measure of cluster separation. Panel (B) shows the clustering on the first two principal components. The red color represents enterotype 1 (*Bacteroides*), and the green color represents enterotype 2 (*Prevotella*). (b) Abundance index of *Bacteroides* and *Prevotella* in each enterotype. Boxes represent the interquartile range (IQR), and the line inside represents the median. PCoA: principal coordinates analysis. (c) LDA EffectSize analysis of two enterotypes. The *x*-axis shows the LDA score (log 10) after analysis and the *y*-axis shows the significantly differential genus between two enterotypes. The LDA threshold value is 2.

All 171 subjects were divided into two clusters with the strongest support (Figure 3(a)). One cluster was the *Bacteroides* enterotype (ET B), which included 134 subjects (21 controls, 113 T2Ds), and another was the *Prevotella* enterotype (ET P), which included 37 subjects (16 controls, 21 T2Ds) (Figure 3(b), [Supplementary-material supplementary-material-1]). ET B was enriched with 16 genera, including *Bacteroides*, *Bifidobacterium*, *Clostridium XIVa*, *Parabacteroides*, *Staphylococcus*, *Granulicatella*, *Porphyromonas*, *Clostridium XI*, *Blautia*, *Anaerostipes*, *Clostridium XVIII*, *Fusicatenibacte*r, *Enterococcus*, *Clostridium IV*, *Eggerthella*, and *Flavonifractor*. In turn, ET P was enriched with three genera, including *Prevotella*, *Dialister*, and *Sutterella* (Figure 3(c)). For ET B, the relative abundances of the genera *Eggerthella*, *Enterocuccus*, *Granulicatella*, and *Bifidobacterium* were significantly different between the T2D and control groups and all of them were higher in the T2D group. For ET P, the relative abundance of the genera *Prevotella* was significantly different between the two groups, and it was higher in the control group ([Supplementary-material supplementary-material-1]).

Antidiabetic drugs taken by T2D patients were compared between the two enterotypes. We found that there was no significant difference from drug usage in the two enterotypes ([Supplementary-material supplementary-material-1]).

#### 3.3.2. Correlation Analysis of Differential Genus and Glucose Metabolic Indices

Among the genera abundant in the two enterotypes, some were associated with glucose metabolic indices. For ET P, *Prevotella* had a negative correlation with HbA1c, FPG, 2hPG, HOMA2-IR, and a positive correlation with Gutt-ISI (*r*_1_ = −0.187, *r*_2_ = −0.284, *r*_3_ = −0.252, *r*_4_ = −0.289, *r*_5_ = 0.296, all *P* < 0.05). For ET B, *Enterococcus* and *Granulicatella* had a positive correlation with HbA1c (*r*_1_ = 0.237, *r*_2_ = 0.254, *P* < 0.05). *Eggerthella* had a positive correlation with FPG (*r* = 0.189, *P* = 0.034). *Eggerthella*, *Enterococcus*, and *Bifidobacterium* had a positive correlation with 2hPG (*r* = 0.291, *r*_1_ = 0.236, *r*_2_ = 0.242, all *P* < 0.05). *Eggerthella* had a negative correlation with HOMA2-*β* (*r* = −0.209, *P* = 0.019) while *Clostridium XVIII* had a positive correlation with HOMA2-*β* (*r* = 0.202, *P* = 0.024). *Bacteroides*, *Bifidobacterium*, and *Fusicatenibacter* had a positive correlation with HOMA2-IR (*r*_1_ = 0.178, *r*_2_ = 0.207, *r*_3_ = 0.215, all *P* < 0.05). *Eggerthella*, *Enterococcus*, *Granulicatella*, and *Bifidobacterium* had a negative correlation with Gutt-ISI (*r*_1_ = −0.363, *r*_2_ = −0.231, *r*_3_ = −0.218, *r*_4_ = −0.299, all *P* < 0.05) ([Supplementary-material supplementary-material-1]).

#### 3.3.3. Logistic Regression Analysis of the Association of Enterotypes with T2D

We established three different models to observe the associations of enterotypes with T2D (Table 2). In model 1, we first simply addressed the role of enterotypes in T2D and found that ET B presented a significantly increased odds ratio (OR) for T2D (OR = 4.100, *P* = 0.001). Then, we adjusted model 1 for gender, age, and BMI to produce model 2 and observed that ET B had the same effect on T2D (OR = 4.362, *P* = 0.001). Additionally, both age and male gender were risk factors for T2D; however, BMI had no relationship with T2D. To exclude the effects of other factors on the results, we further adjusted model 2 for TG and HDL-C (model 3). Interestingly, ET B and age still significantly increased the risk for T2D (OR_1_ = 3.124, *P*_1_ = 0.029; OR_2_ = 1.066, *P*_2_ = 0.003), while gender and BMI had no effect. Furthermore, TG was also a risk factor for T2D, while HDL-C was a protective factor. To further explore the role of enterotypes in T2D, we added HOMA-*β*, HOMA-IR, and Gutt-ISI in the logistic regression to model 3, thus establishing models 4, 5, and 6, respectively (Table 2). Surprisingly, when HOMA-*β* and HOMA-IR were added to the analysis, the ET B still had a significant effect on T2D; however, when Gutt-ISI was added, the ET B no longer exhibited a significant influence on T2D (*P* = 0.059).

#### 3.3.4. Comparison of HOMA-*β*, HOMA-IR, and Gutt-ISI between the Two Enterotypes

Based on the different results regarding the relationship between ET B and T2D in models 3 and 6 (Table 2), we reasoned that ET B might increase the risk of developing T2D by decreasing insulin sensitivity. To further verify this assumption, we compared the HOMA-*β*, HOMA-IR, and Gutt-ISI values between the two enterotypes ([Supplementary-material supplementary-material-1]), which suggested that there was a difference in insulin sensitivity, but not in *β*-cell function.

#### 3.3.5. Comparison of DAO, LPS, TNF-*α*, and Age between Two Enterotypes

Previous studies have found that the gut microbiota could decrease insulin sensitivity by endotoxemia and low-grade inflammation [24, 25]. Therefore, we compared the levels of DAO, LPS, and TNF-*α* between ET B and ET P and found that the levels of LPS and TNF-*α* were higher in ET B, while there was no significant difference in DAO level and age (Table 3).

#### 3.3.6. Correlation Analysis of DAO, LPS, TNF-*α*, Age, and Fecal Microbiome

Spearman correlation analysis was used to explore further the relationship between DAO, LPS, TNF-*α*, age, and genus enriched in ET B and ET P. For ET P, *Sutterella* had a negative correlation with age (*r* = −0.234, *P* = 0.009). *Prevotella* had a negative correlation with LPS and TNF-*α* (*r*_1_ = −0.24, *r*_2_ = −0.319, all *P* < 0.05). For ET B, *Bifidobacterium* and *Eggerthella* had a positive correlation with age (*r*_1_ = 0.392, *r*_2_ = 0.256, both *P* < 0.05). *Blautia*, *Eggerthella*, and *Flavonifractor* had a positive correlation with DAO (*r*_1_ = 0.222, *r*_2_ = 0.309, *r*_3_ = 0.241, all *P* < 0.05). *Bifidobacterium*, *Granulicatella*, and *Eggerthella* had a positive correlation with LPS (*r*_1_ = 0.246, *r*_2_ = 0.187, *r*_3_ = 0.223, all *P* < 0.05). *Flavonifractor* had a positive correlation with TNF-*α* (*r* = 0.214, *P* = 0.024) ([Supplementary-material supplementary-material-1]).

#### 3.3.7. Partial Correlation Analysis of DAO, LPS, TNF-*α*, and Gutt-ISI

To further explore the association between the DAO, LPS, TNF-*α* levels, and insulin sensitivity, we analyzed the correlation of Gutt-ISI with DAO, LPS, and TNF-*α* using partial correlation analysis by adjusting for age, BMI, TG, and HDL-C (Table 4). Interestingly, LPS was closely associated with DAO, TNF-*α*, and Gutt-ISI. Moreover, TNF-*α* trended toward a negative correlation with Gutt-ISI (*P* value = 0.059).

#### 3.3.8. Multiple Linear Regression Analysis

We also performed multiple linear regression analysis in order to determine if LPS or DAO levels were independent risk factors for insulin sensitivity (Table 5). We concluded from the results that LPS was an independent risk factor for insulin sensitivity after adjusting for age, BMI, TG, HDL-C, DAO, and TNF-*α* levels. When it comes to DAO, after adjusting for age, it was no longer a risk factor for insulin sensitivity ([Supplementary-material supplementary-material-1]).

## 4. Discussion

In our study, we first compared the differences in the gut microbiome between T2D and control group. Diversity analysis showed that the composition of the microbiota was different between the control and T2D group, and the T2D group had lower bacterial diversity than the control group, findings that are consistent with former studies [1, 2]. The differential analysis showed that the numbers of candidate taxa associated with the gut microbial dysbiosis in the T2D group were 3 at the phylum level and 23 at the genera level. At the phylum level, some studies detected a higher quantity of Bacteroidetes in T2D patients, which seemed inconsistent with our result [1, 8]. Studies showed that the lower prevalence of phylum Bacteroidetes and increased Firmicutes/Bacteroidetes ratio was related to obesity and dyslipidemia [26, 27]. In our study, patients in T2D group had higher TG level than the control group, which might influence the result. At the genus level, most differential genera were consistent with other studies, such as the *Prevotella* enrichment in the control group [8], or *Escherichia/Shigella*, *Lactobacillus* enrichment in the T2D group [28]. However, *Bifidobacterium* was enriched in the T2D group, which might be due to AGI usage [14]. Consistent with one of the mechanisms regarding to the gut microbiome and T2D, we found increased DAO, LPS, and TNF-*α* levels in the T2D group, which showed impaired intestinal permeability, metabolic endotoxemia, and low-grade inflammation in the T2D group.

Enterotype analysis has been proposed as a useful method to understand human microbial communities, including *Bacteroides*, *Ruminococcus*, and *Prevotella* enterotypes, irrespective of ethnicity, gender, age, or BMI [16]. In 2011, Wu et al. clustered human fecal communities into two enterotypes distinguished primarily by the levels of *Bacteroides* and *Prevotella*. Then, they found that these two enterotypes were strongly associated with long-term diet, particularly protein and animal fat (*Bacteroides*) versus carbohydrates (*Prevotella*). In our study, all fecal samples were divided into the same two enterotypes, although there was no dietary difference among individuals. A differential analysis between two enterotypes and a correlation analysis of differential genus and glucose metabolic indexes showed that there was a relationship between the enterotype and T2D. Further logistic regression analysis revealed that ET B was an independent risk factor for T2D, which may be mediated by the decrease in insulin sensitivity rather than an effect on *β*-cell function. Based on previous studies regarding the gut microbiota and insulin resistance [24, 25], we assumed that the ET B was associated with increased intestinal permeability, which then resulted in a higher bacterial LPS concentration in the blood. Endotoxemia-induced low-grade inflammation can cause a decrease in insulin sensitivity. Therefore, we compared the levels of DAO, LPS, and TNF-*α* in two enterotypes and found that the ET B group had higher LPS and TNF-*α* levels but no significant change in DAO level. Further correlation analysis showed the genera *Eggerthella*, which had the strongest positive correlation with DAO, also had a positive correlation with age. Thus, compared to enterotype, age might play a more important role in the DAO level, and the higher level of LPS in ET B might be independent of DAO in our subjects. Partial correlation analysis adjusting for age, BMI, TG, and HDL-C showed LPS was positively correlated with DAO and TNF-*α* and negatively correlated with insulin sensitivity. That explained why age was an independent risk factor for T2D. After adjusting for age, BMI, TG, HDL-C, DAO, and TNF-*α*, the level of LPS was still an independent risk factor for insulin sensitivity. But after adjusting for age, DAO was no longer a risk factor for Gutt-ISI. Based on the results above, we concluded that the higher DAO found in the T2D group was the result of older age and that people with ET B exhibited a higher concentration of serum LPS independent of DAO. This increase in serum LPS levels has the potential to cause endotoxemia and low-grade inflammation, which could, in turn, decrease insulin sensitivity.

Previous studies found that ET P was related to a high fiber diet [19] and the dietary fiber-induced improvement in glucose metabolism was associated with an increased abundance of *Prevotella* and a high ratio of *Prevotella*/*Bacteroides* [11]. Rampelli et al. found that the functional repertoire of *Prevotella* was linked to an increased capacity of the gut microbiota to ferment complex polysaccharides from the diet [29].Those studies suggested that the different capacity of *Prevotella* and *Bacteroides* to perform carbohydrate fermentation in the gut contributed to the differences observed in terms of glucose metabolism and highlighted the importance of Prevotella in improving glucose tolerance. The beneficial mechanism is still not clear. In our study, we also observed that ET P was a protective factor for the glucose metabolism. Although there was no difference in diet, ET P, which had higher *Prevotella*, could utilize carbohydrates in the diet more efficiently and might produce more SCFA. SCFA is a major energy source for enterocyte and could help to maintain the intestinal wall integrity.

For ET B, although *Bacteroides* was a drive genera, its abundance was not different between T2D and control group, and it had no correlation with factor levels. So other genera might play a predominant role on insulin resistance. Besides *Bacteroides*, ET B was enriched with many opportunistic pathogens such as *Eggerthella*, *Clostridium*, *Staphylococcus*, *Granulicatella*, and *Enterococcus*. Many of them were also found enriched in T2D patients in other studies [1, 3]. Those genera may damage intestinal permeability by endotoxin or exotoxin, which then leads to bacterial translocation, endotoxemia, low-grade inflammation, and insulin resistance.

Our study suggested that ET B was a risk factor for T2D because of endotoxemia and low-grade inflammation. After analyzing the characteristics of two enterotypes, we thought that the higher LPS level in ET B might due to damaged intestinal permeability. But our study did not find a significantly increased DAO level in ET B. A few reasons may explain this scenario. Firstly, the intestinal barrier function comprises three major parts: a mechanical barrier, an ecological barrier, and an immunological barrier. DAO is just a biomarker of intestinal epithelial integrity, which could partly reflect the mechanical barrier function. Since other barrier functions, such as tight junction structural function and the mucous immunological function, were not evaluated, further research should be done to compare the intestinal permeability between ET B and ET P enterotype. Secondly, the sample number of two enterotypes was unbalanced, which affect the Mann–Whitney *U* test result.

Our study has some limitations. First, the number of samples analyzed was small, which means that the representativeness of our study was not ideal. Second, we only utilized one method to cluster the fecal samples, and we did not confirm the microbiota structure in other populations. Third, the design of our study was cross-sectional, which cannot prove cause-and-effect relationships. Based on the above, the exact effect enterotypes have on T2D needs to be further verified by a large prospective study and also needs to be confirmed in animal models. Nonetheless, the results from our study provide a new direction through which to explore the relationship between the gut microbiota and T2D.

## 5. Conclusions

Our study aimed at exploring the existence of enterotypes among patients with T2D and nondiabetic controls and if there is a relationship between the enterotype and T2D. Our results demonstrated that there was a significant difference in the gut microbiota structure between the T2D and nondiabetic groups. Furthermore, we identified two enterotypes among our subjects and showed that ET B was an independent risk factor for T2D. Moreover, this increased risk was associated with increased LPS levels and decreased insulin sensitivity in the ET B, which was independent of the DAO level but might be related to the intestinal barrier function. Taken together, our study provides new insights into the relationship between the gut microbiota and T2D, and a new direction through which to research the interaction between T2D and the gut microbiota.

## Figures and Tables

**Table 1:** Characteristics of T2D and control groups.

Parameters	Control group	T2D group	*P* value
Age (year)^∗^	50.00 (45.00, 57.50)	59.50 (50.27, 66.64)	<0.001
BMI (kg/m^2^)	25.84 (23.10, 27.17)	25.39 (23.49, 27.51)	0.896
SBP (mmHg)	133 (120, 147)	131.25 (123.00, 145.50)	0.912
DBP (mmHg)^∗^	81.80 ± 11.31	75.85 ± 10.52	0.004
HbA1c (%)^∗^	5.10 (4.90, 5.40)	8.40 (7.30, 9.70)	<0.001
FPG (mmol/L)^∗^	5.30 (5.20, 5.50)	7.57 (6.31, 8.95)	<0.001
FINS (uIU/ml)^∗^	6.85 (4.29, 8.99)	10.72 (5.97, 19.77)	<0.001
FCP (ng/ml)	1.19 (0.96, 1.58)	1.21 (0.86, 2.01)	0.535
2hPG (mmol/L)^∗^	5.60 (5.23, 7.45)	16.96 (14.60, 18.41)	<0.001
2hINS (uIU/ml)^∗^	20.81 (15.53, 35.11)	38.40 (22.63, 54.57)	<0.001
2hCP (ng/ml)^∗^	4.62 (4.08, 8.00)	3.87 (2.56, 5.39)	0.001
HDL-C (mmol/L)^∗^	1.55 (1.38, 1.85)	1.20 (1.02, 1.41)	<0.001
TG (mmol/L)^∗^	1.04 (0.77, 1.38)	1.38 (1.00, 1.92)	0.001
Gutt-ISI∗	0.54 (0.42, 0.65)	0.20 (0.17, 0.25)	<0.001
HOMA-*β* (CP)^∗^	77.00 (61.70, 90.50)	41.45 (26.60, 63.75)	<0.001
HOMA-IR (CP)	0.89 (0.74, 1.19)	1.05 (0.70, 1.68)	0.100

The normal distribution of the data was tested using the Kolmogorov-Smirnov test. Values are expressed as median (interquartile range; abnormal distribution) and mean ± SD (normal distribution). Differences between groups were detected using a Student's *t* test (normal distribution) or Mann–Whitney *U* test (abnormal distribution). T2D: type 2 diabetes; BMI: body mass index; SBP: systolic blood pressure; DBP: diastolic blood pressure; HbA1c: glycated hemoglobin; FPG: fasting plasma glucose; FINS: fasting insulin; FCP: fasting C peptide; 2hPG: 2-hour postprandial plasma glucose; 2hINS: 2-hour postprandial plasma insulin; 2hCP: 2-hour postprandial C peptide; HDL-C: high-density lipoprotein cholesterol; TG: triglyceride levels; Gutt-ISI: Gutt-insulin sensitivity index; HOMA-*β*: homeostasis model assessment of *β*-cell function; HOMA-IR: homeostasis model assessment of insulin resistance. ^∗^*P* < 0.05.

**(a):** 

Characteristics	Model 1	*P* value	Model 2	*P* value	Model 3	*P* value
	OR (95% CI)		OR (95% CI)		OR (95% CI)	
ET B	4.100 (1.842–9.124)	0.001^∗^	4.362 (1.820–10.454)	0.001^∗^	3.124 (1.121–8.705)	0.029^∗^
Male	—	—	3.002 (1.268–7.109)	0.012^∗^	1.218 (0.435–3.408)	0.708
Age (years)	—	—	1.057 (1.022–1.093)	0.001^∗^	1.066 (1.023–1.111)	0.003^∗^
BMI (kg/m^2^)	—	—	1.006 (0.909–1.113)	0.911	0.928 (0.830–1.037)	0.188
TG (mmol/l)	—	—			1.604 (1.030–2.497)	0.037^∗^
HDL-C (mmol/l)	—	—			0.038 (0.008–0.184)	<0.001^∗^

**(b):** 

Characteristics	Model 4	*P* value	Model 5	*P* value	Model 6	*P* value
	OR (95% CI)		OR (95% CI)		OR (95% CI)	
ET B	4.668 (1.298–16.786)	0.018^∗^	3.096 (1.096–8.742)	0.033^∗^	5.786 (0.935–35.801)	0.059
Male	1.871 (0.560–6.254)	0.309	1.218 (0.434–3.416)	0.708	2.141 (0.371–12.347)	0.394
Age (years)	1.059 (1.006–1.114)	0.029^∗^	1.065 (1.021–1.110)	0.003^∗^	1.070 (1.001–1.144)	0.047^∗^
BMI (kg/m^2^)	0.918 (0.810–1.041)	0.183	0.929 (0.828–1.043)	0.047^∗^	—	**—**
TG (mmol/l)	1.760 (1.030–3.006)	0.038^∗^	1.612 (1.032–2.518)	0.036^∗^	0.915 (0.658–1.273)	0.915
HDL-C (mmol/l)	0.014 (0.002–0.107)	<0.001^∗^	0.037 (0.007–0.191)	<0.001^∗^	0.170 (0.016–1.808)	0.170
HOMA-*β*	0.956 (0.938–0.974)	<0.001^∗^	—	—	—	—
HOMA-IR	—	—	0.903 (0.386–2.112)	0.814	—	—
Gutt-ISI	—	—	—	—	0.000 (0.000–0.000)	<0.001^∗^

Model 1 had no adjusted variable. Model 2 is adjusted for age, gender, and BMI. Model 3 is adjusted for model 2 plus TG and HDL-C. Model 4 is adjusted for model 3 plus HOMA-*β*. Model 5 is adjusted for model 3 plus HOMA-IR. Model 6 is adjusted for model 3 plus Gutt-ISI, since body weight was used to calculate Gutt-ISI, BMI was removed in Model 6. ^∗^*P* < 0.05. ET B: enterotype *Bacteroides*; BMI: body mass index; TGs: triglyceride levels; HDL-C: high-density lipoprotein cholesterol; HOMA-*β*: homeostasis model assessment of *β*-cell function; HOMA-IR: homeostasis model assessment of insulin resistance; Gutt-ISI: Gutt-insulin sensitivity index.

**Table 3:** Comparison of the four factors in the ET B and ET P.

Factor	ET B	ET P	*P* value
DAO (mIU/ml)	367.75 (223.31, 547.55)	258.50 (167.01, 404.86)	0.081
LPS (pg/ml)	128.88 (89.04, 184.00)	88.73 (64.81, 128.83)	0.007^∗^
TNF-*α* (pg/ml)	58.28 (35.81, 93.76)	36.75 (22.01, 87.43)	0.047^∗^
Age (years)	58.61 (49.60, 65.24)	52.44 (47.00, 63.82)	0.188

^∗^ *P* < 0.05. ET B: enterotype *Bacteroides*; ET P: enterotype *Prevotella*; DAO: diamine oxidase; LPS: lipopolysaccharide; TNF-*α*: tumor necrosis factor-alpha.

**Table 4:** Partial correlation analysis of Gutt-ISI with DAO, LPS, and TNF-*α*.

Characteristic	DAO	*P*	LPS	*P*	TNF-*α*	*P*	Gutt-ISI	*P*
	*r*		*r*		*r*		*r*	
DAO			0.237	0.006^∗^	0.094	0.279	-0.032	0.717
LPS	0.237	0.006^∗^			0.242	0.005^∗^	-0.223	0.010^∗^
TNF-*α*	0.094	0.279	0.242	0.005^∗^			-0.164	0.059
Gutt-ISI	-0.032	0.717	-0.223	0.010^∗^	-0.164	0.059		

Partial correlation analysis was adjusted for age, BMI, TG, and HDL-C. *r*: correlation coefficient; *P*: *P* value ^∗^*P* < 0.05; DAO: diamine oxidase; LPS: lipopolysaccharide; TNF-*α*: tumor necrosis factor-alpha; Gutt-ISI: Gutt-insulin sensitivity index.

**Table 5:** Multiple linear regression analysis of the association of Gutt-ISI with LPS.

Characteristics	Model 1	*P* value	Model 2	*P* value	Model 3	*P* value
	B		B		B	
LPS (pg/ml)	0.000 (-0.001-0.000)	<0.001^∗^	0.000 (-0.001-0.000)	0.010^∗^	0.000 (-0.001-0.000)	0.027^∗^
Age (years)	—	**—**	-0.005 (-0.007-0.002)	0.001^∗^	-0.005 (-0.008-0.002)	0.001^∗^
BMI (kg/m^2^)	—	—	-0.001 (-0.009-0.007)	0.787	-0.002 (-0.010-0.007)	0.702
TG (mmol/l)	—	**—**	-0.020 (-0.033-0.006)	0.005^∗^	-0.020 (-0.034-0.006)	0.005^∗^
HDL-C (mmol/l)	—	**—**	0.180 (0.096-0.264)	<0.001^∗^	0.179 (0.095-0.263)	<0.001^∗^
DAO (mIU/ml)	—	**—**	—	—	0.000 (0.000-0.000)	0.758
TNF-*α* (pg/ml)	—	—			0.000 (-0.001-0.000)	0.182

Model 1 had no adjusted variable. Model 2 is adjusted for age, BMI, TG, and HDL-C. Model 3 is adjusted for model 2 plus DAO and TNF-*α*. *P*: *P* value, ^∗^*P* < 0.05; Gutt-ISI: Gutt-insulin sensitivity index; DAO: diamine oxidase; LPS: lipopolysaccharide; TNF-*α*: tumor necrosis factor-alpha; BMI: body mass index; TG: triglyceride levels; HDL-C: high-density lipoprotein cholesterol.

## Data Availability

The data used to support the findings of this study are included within the supplementary information file(s).

## Supplementary Materials

Supplementary 1 Table S1. Number of clean reads and average length of reads in each subject.

Supplementary 2 Table S2. Distribution of the sequence length of trimmed reads.

Supplementary 3 Table S3. Number of OTUs (operational taxonomic units) in each subject.

Supplementary 4 Table S4. Final number of OTUs of each subject after equalizing.

Supplementary 5 Table S5. The number of OTUs that can be assigned at each level.

Supplementary 6 Table S6. Distribution of OTUs in 16 bacterial phyla in each sample.

Supplementary 7 Table S7. Analysis of the *α*-diversity in the control and T2D groups.

Supplementary 8 Table S8. Differential genera between T2D and control group.

Supplementary 9 Table S9. Comparison of three factors between T2D and control group.

Supplementary 10 Table S10. Cluster to which each sample belongs.

Supplementary 11 Table S11. Drug usage in the two enterotypes.

Supplementary 12 Table S12. Correlation analysis of differential genus and glucose metabolic indices.

Supplementary 13 Table S13. Comparison of the two enterotypes using three indices.

Supplementary 14 Table S14. Correlation analysis of DAO, LPS, TNF-*α*, age, and fecal microbiome.

Supplementary 15 Table S15. Multiple linear regression analysis of the association of Gutt-ISI with DAO.

Supplementary 16 Figure S1. Dilution curve image of the Chao1 index, length of trimmed reads, and OTU Venn diagram of all subjects.
