# Supplementary material for: Enterotype Bacteroides Is Associated with a High Risk in Patients with Diabetes: A Pilot Study
Source: J Diabetes Res. 2020 Jan 22;2020:6047145. doi: 10.1155/2020/6047145 (PMC6996672; doi:10.1155/2020/6047145)
Supplement: Supplementary 8 — Table S8. Differential genera between T2D and control group. [file 6047145.f7.docx]

**Table S7. Analysis of the α-diversity in the control and T2D groups.**

| Alpha name | Observed species index | Shannon index |
| --- | --- | --- |
| Mean (Control group) | 193.81 | 4.48 |
| Mean (T2D group) | 171.99 | 4.06 |
| P-value | 0.040^＊^ | 0.009^＊^ |

The first row is the α-diversity indices. The second and third rows are the mean values of the two indices in the control and T2D groups. The last row is the P-value of the two indices among the two groups using a rank sum test.
