# Supplementary material for: Enterotype Bacteroides Is Associated with a High Risk in Patients with Diabetes: A Pilot Study
Source: J Diabetes Res. 2020 Jan 22;2020:6047145. doi: 10.1155/2020/6047145 (PMC6996672; doi:10.1155/2020/6047145)
Supplement: Supplementary 10 — Table S10. Cluster to which each sample belongs. [file 6047145.f9.docx]

**Table S11. Comparison of three factors between T2D and control group**

| Factor | CON | T2D | P value |
| --- | --- | --- | --- |
| DAO | 290.20(141.81,411.03) | 370.90(233.67,575.81) | 0.015^＊^ |
| LPS | 73.08(54.52,95.90) | 137.51(100.45,197.83) | <0.001^＊^ |
| TNF-α | 36.28(17.49,82.31) | 58.10(37.03,93.95) | 0.038^＊^ |

^＊^P<0.05. CON: control group. T2D: type 2 diabetes group. DAO: diamine oxidase. LPS: lipopolysaccharide. TNF-α: tumor necrosis factor-alpha.
