# Supplementary material for: Enterotype Bacteroides Is Associated with a High Risk in Patients with Diabetes: A Pilot Study
Source: J Diabetes Res. 2020 Jan 22;2020:6047145. doi: 10.1155/2020/6047145 (PMC6996672; doi:10.1155/2020/6047145)
Supplement: Supplementary 11 — Table S11. Drug usage in the two enterotypes. [file 6047145.f11.docx]

**Table S11. Drug usage in the two enterotypes.**

| Drug | ET B | ET P | P-value |
| --- | --- | --- | --- |
| metformin | 86 | 18 | 0.406 |
| AGI | 59 | 12 | 0.813 |
| Insulin | 69 | 16 | 0.224 |
| Statins | 25 | 8 | 0.165 |

ET B: enterotype *Bacteroides.* ET P: enterotype *Prevotella.* AGI:ɑ-glucosidase inhibitors
