# Supplementary material for: Enterotype Bacteroides Is Associated with a High Risk in Patients with Diabetes: A Pilot Study
Source: J Diabetes Res. 2020 Jan 22;2020:6047145. doi: 10.1155/2020/6047145 (PMC6996672; doi:10.1155/2020/6047145)
Supplement: Supplementary 13 — Table S13. Comparison of the two enterotypes using three indices. [file 6047145.f13.docx]

**Table S10.** **Comparision of the two enterotypes using three indices**

| Index | ET B | ET P | P-value |
| --- | --- | --- | --- |
| HOMA-β | 47.15(31.70, 75.50) | 56.65(38.33, 76.68) | 0.319 |
| HOMA-IR | 1.09(0.70, 1.65) | 0.90(0.73, 1.94) | 0.066 |
| Gutt-ISI | 0.22(0.18, 0.29) | 0.28(0.20, 0.55) | 0.013^＊^ |

^＊^P<0.05. ET B: enterotype *Bacteroides.* ET P: enterotype *Prevotella.* HOMA-β: Homeostasis model assessment of β-cell function. HOMA-IR: homeostasis model assessment of insulin resistance. Gutt-ISI: Gutt-insulin sensitivity index.
