# Supplementary material for: Enterotype Bacteroides Is Associated with a High Risk in Patients with Diabetes: A Pilot Study
Source: J Diabetes Res. 2020 Jan 22;2020:6047145. doi: 10.1155/2020/6047145 (PMC6996672; doi:10.1155/2020/6047145)
Supplement: Supplementary 15 — Table S15. Multiple linear regression analysis of the association of Gutt-ISI with DAO. [file 6047145.f15.docx]

| Characteristics | Model 1 |  |  | Model 2 |  |  |
| --- | --- | --- | --- | --- | --- | --- |
|  | B | P-value |  | B | P-value |  |
| DAO (mIU/ml) | 0.000(0.000-0.000) | 0.036^＊^ |  | 0.000(0.000-0.000) | 0.323 |  |
| Age (years) | - | **-** |  | -0.005(-0.008--0.002) | 0.002^＊^ |  |

**Table S15. Multiple linear regression analysis of the association of Gutt-ISI with DAO**
