# Supplementary figures and images for: Enterotype Bacteroides Is Associated with a High Risk in Patients with Diabetes: A Pilot Study
Source: J Diabetes Res. 2020 Jan 22;2020:6047145. doi: 10.1155/2020/6047145 (PMC6996672; doi:10.1155/2020/6047145)

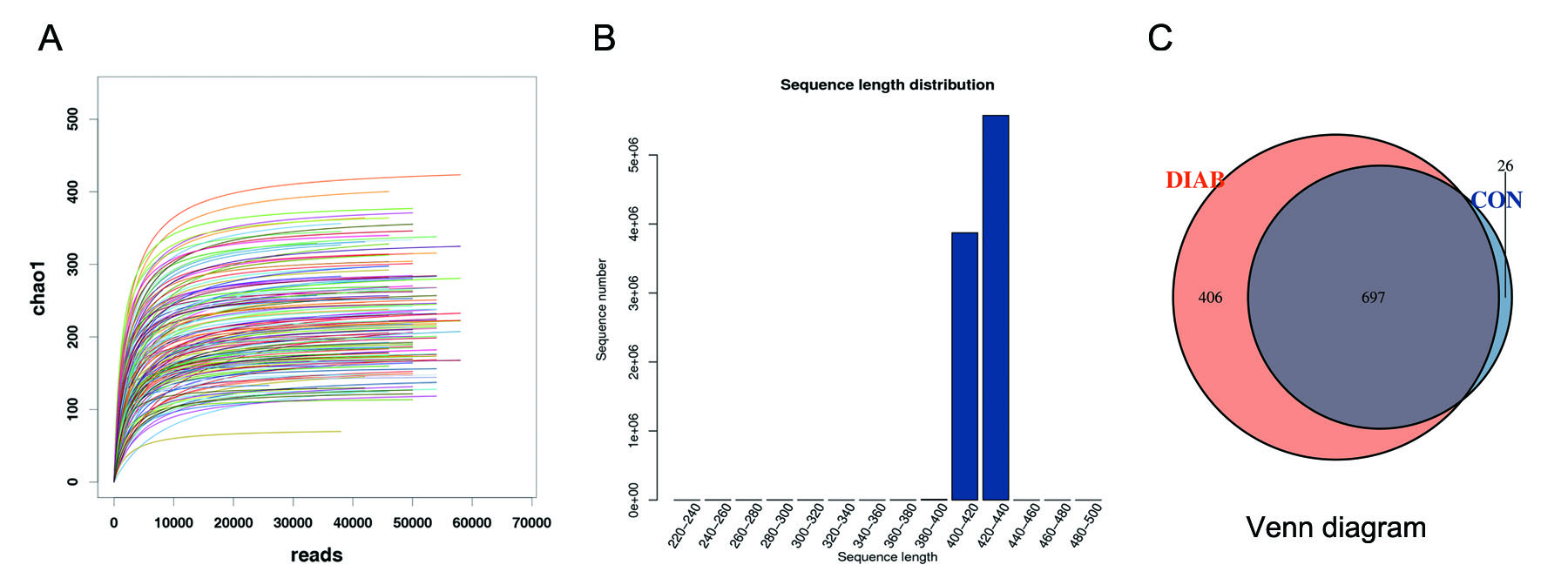

Supplement: Supplementary 16 — Figure S1. Dilution curve image of the Chao1 index, length of trimmed reads, and OTU Venn diagram of all subjects. [file 6047145.f16.jpg]
